# Management and analysis of complications associated with all‐inside technique anterior cruciate ligament reconstruction: A propensity score‐matched study

**DOI:** 10.1002/jeo2.70569

**Published:** 2026-01-08

**Authors:** Yang Tang, Dongxu Yan, Liang Xu, Di Wu, Jingyu Gao, Yuan Wu, Gang Yu, Chao Fang, Qichun Zhao

**Affiliations:** ^1^ Department of Orthopedics The First Affiliated Hospital of USTC, Division of Life Sciences and Medicine, University of Science and Technology of China Hefei Anhui China; ^2^ Division of Life Sciences and Medicine University of Science and Technology of China Hefei Anhui China

**Keywords:** all‐inside technique, anterior cruciate ligament reconstruction, arthroscopy, complications, clinical outcome

## Abstract

**Purpose:**

To investigate the incidence and management of complications associated with all‐inside technique (AIT) anterior cruciate ligament reconstruction (ACLR), and to compare postoperative outcomes between patients with successfully managed complications and complication‐free controls.

**Methods:**

A retrospective analysis was conducted on patients undergoing AIT‐ACLR. AIT‐related complications were documented, with a minimum 24‐month follow‐up. Propensity score matching (PSM, 1:2 ratio) was used to compare the complication and non‐complication groups. Knee function was assessed using the Lysholm Knee Score, International Knee Documentation Committee Subjective Score, and Tegner Activity Scale. Stability was measured with the Ligs Digital Arthrometer.

**Results:**

A total of 274 patients were included, with 45 patients (16.4%) experiencing AIT‐related complications. Complications comprised tibial lateral subluxation (21 cases, 7.7%; mean displacement: 2.3 mm, range 2.0–2.5 mm), which resolved spontaneously in 4 patients (19.1%) by 1 month postoperatively and in the remaining cases by 3 months. Femoral suspensory button malposition occurred in 12 patients (4.4%), with only one case (8.3%) requiring immediate revision due to a 7.6 mm displacement. The others (mean displacement: 2.5 mm, range 2.1–3.0 mm) were managed conservatively. Cortical breach at the tibial tunnel exit (7 cases, 2.6%) and flip drill bit breakage (5 cases, 1.8%) were addressed intraoperatively. Following PSM (complication group: *n* = 45 vs. non‐complication group: *n* = 87), baseline demographics demonstrated no significant differences except for operative time (*p* = 0.035). There were no statistically significant differences in knee function and stability between the matched groups at 3, 6, 12, and 24 months postoperatively (*p* > 0.05 for all).

**Conclusion:**

Postoperative knee function and stability demonstrated improvement following AIT‐ACLR. Although appropriately managed complications did not substantially compromise clinical outcomes, the findings emphasize the importance of technical vigilance, intraoperative complication management, and preventive strategies to optimize surgical outcomes.

**Level of Evidence:**

Level III, case‐control study.

**Abbreviations**
3D‐CT: three‐dimensional computed tomography
80N12: 80 N at 12 months after surgery
80N24: 80 N at 24 months after surgery
ACLR: anterior cruciate ligament reconstruction
AIT: all‐inside technique
AP: anteroposterior
Bit: breakage of the flip drill bit
BMI: body mass index
Bre: cortical breach at the tibial tunnel exit
CI: confidence interval
Com: complication
Con: control
G: gracilis
IKDC: International Knee Documentation Committee
Mal: femoral suspensory button malposition
Non‐com: non‐complication
PSM: propensity score matching
SMD: standardized mean difference
SSD: side‐to‐side differences
ST: semitendinosus
Sub: tibial lateral subluxation

## INTRODUCTION

Anterior cruciate ligament (ACL) injuries are increasingly common [3, 12, 38]. Untreated ACL injuries impair knee function, destabilize joints, and elevate risks of post‐traumatic osteoarthritis [15, 30]. Arthroscopic ACL reconstruction (ACLR) remains the primary surgical intervention [1, 13]. Surgical techniques for ACLR vary significantly in terms of graft type, tunnel positioning, and fixation methods [2]. Among these, the all‐inside technique (AIT) offers advantages, especially for tibial‐sided fixation [16]. Compared to traditional techniques, AIT offers several advantages, including shorter bone tunnels, reduced surgical trauma, preservation of autograft tendons, minimized bone loss, and enhanced graft fixation strength [4, 5, 31].

Despite the distinct advantages of AIT over conventional approaches, complications cannot be entirely avoided. While several studies have reported complications associated with AIT, few studies have systematically analysed AIT‐related complications and their targeted management strategies, particularly regarding innovative approaches to address tibial tunnel cortical breaches [18, 29]. Furthermore, limited literature exists comparing the impact of AIT‐related complications (e.g., tibial lateral subluxation) on postoperative knee function and stability, underscoring a critical gap in understanding clinical implications.

This retrospective cohort study aimed to investigate the incidence and management of complications associated with AIT‐ACLR. Propensity score matching (PSM) was utilized to compare knee function and stability between the complication and non‐complication groups. Additionally, this study describe and analyse the occurrence of complications and the application of their management methods, synthesize and expand on targeted management strategies for complications, and thereby optimize the clinical efficacy of the AIT‐ACLR procedure.

## METHODS

### Patients and inclusion/exclusion criteria

This study was approved by the local medical research ethics committee (Approval No. 2024‐RE‐276). All patients provided written informed consent, which included consent for the use of their anonymized clinical data for research purposes. A retrospective analysis was conducted on patients who underwent AIT‐ACLR at a single institution between January 2022 and April 2023. Patients included in this study were preoperatively diagnosed with ACL rupture through clinical physical assessment and imaging examination. All patients underwent AIT‐ACLR using autograft tendons for reconstruction. The exclusion criteria for this study included the following: (1) age <18 years; (2) multiple ligament injuries; (3) revision ACL surgery; (4) history of knee fractures, tumours, arthritis, or prior surgical procedures; (5) severe polytrauma, neuromuscular disorders, or psychiatric conditions; (6) incomplete clinical data. Although AIT allows for physeal‐sparing ACLR in skeletally immature patients, this specific population was excluded from the current study.

### Surgical techniques and rehabilitation

**Figure 1:**
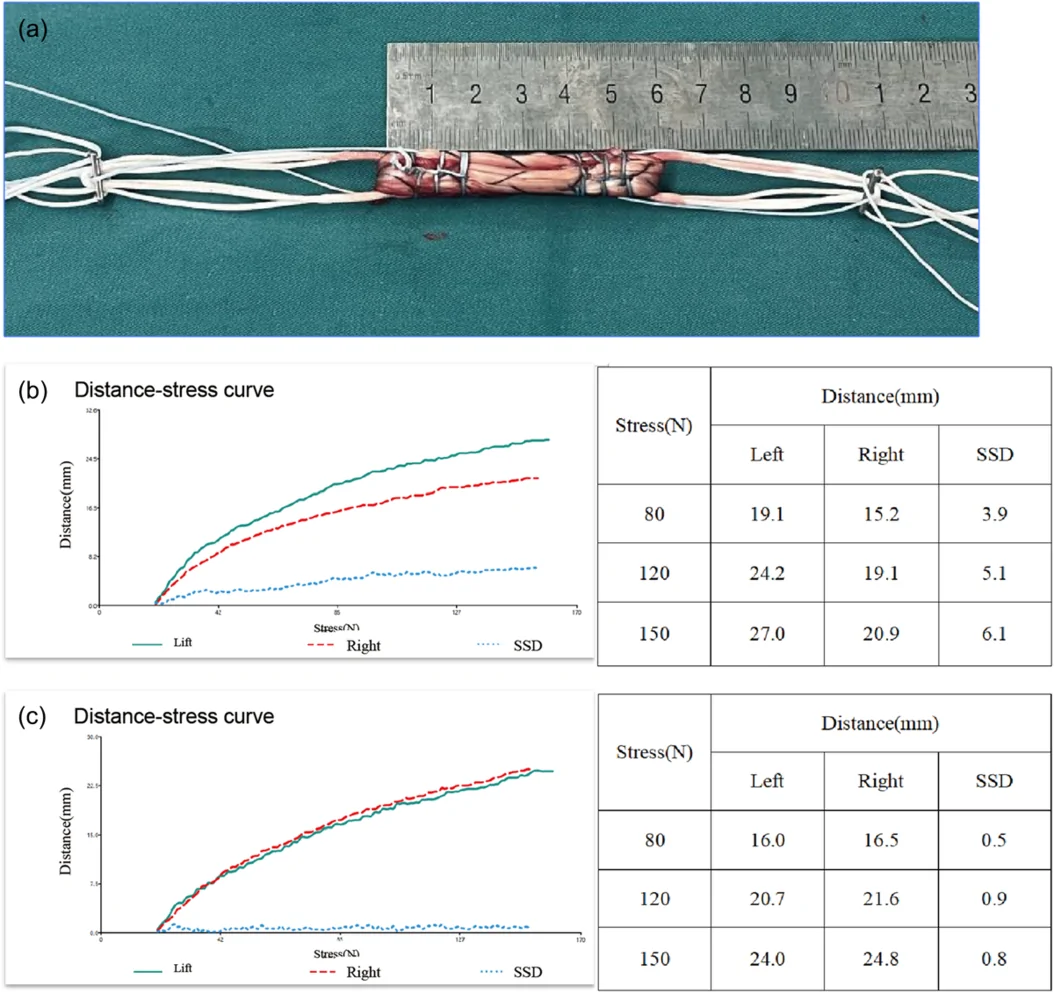
Graft preparation and knee laxity assessment. (a) Prepared autologous tendon grafts with adjustable‐loop suspensory buttons. (b, c) Representative anterior tibial translation curves measured using the Ligs Digital Arthrometer (b) before and (c) after AIT‐ACLR. Side‐to‐side differences (SSD) were calculated under 80, 120, and 150 N loads. The postoperative curve shows reduced tibial translation, indicating restored knee stability. ACLR, anterior cruciate ligament reconstruction; AIT, all‐inside technique; SSD, side‐to‐side differences.

All surgeries were performed by a senior chief physician in sports medicine following a standardized AIT protocol. After establishing the standard anterolateral and anteromedial portals, arthroscopic exploration was conducted, and synovial hyperplasia was debrided to optimize visualization of critical anatomic landmarks, prevent potential graft impingement, and reduce intra‐articular inflammation. For concomitant meniscal injuries, meniscectomy and/or suturing were performed as deemed appropriate. In cases with significant narrowing of the intercondylar notch, intercondylar notch plasty was carried out. The graft was harvested through a longitudinal incision medial to the tibial tuberosity of the affected limb, typically consisting of the semitendinosus tendon. However, in some patients with a poor quality of the semitendinosus tendon that did not meet reconstruction criteria after weaving, additional harvesting of the gracilis tendon was required. The harvested tendons were prepared using the standard GraftLink technique [20]. The grafts were passed through two adjustable‐loop suspensory buttons (FixButton Adjustable Loop, Delta, China), then reinforced and marked with high‐strength sutures (#2 OrthoCord Suture, Johnson & Johnson, USA) [19]. The final length of the completed tendon graft should be ≤75.0 mm [8, 24, 25], and its diameter should be ≥8.0 mm [9, 33] (Figure 1a).

The femoral tunnel was positioned at the I.D.E.A.L. point on the medial aspect of the lateral femoral condyle [28, 34]. A guide pin was inserted, followed by drilling of a femoral socket matched to the graft diameter using a hollow drill. A 4.5‐mm hollow drill was then used to complete the tunnel. For tibial tunnel preparation, the tip of the tibial aimer was positioned at the centre of the ACL remnant, between the anterior horn of the lateral meniscus and the medial tibial spine, and 5–7 mm anterior to the PCL. The aimer angle was typically set within a range of 55° to 60° to ensure an adequate bone bridge. The specific angle within this range was selected to achieve a tunnel length that fulfilled two critical requirements: the required tibial‐sided graft depth and the preservation of a minimum 7 mm narrow tunnel segment [21]. A guidewire was advanced through the medial incision used for tendon harvesting, maintaining the set angle. A 4.5 mm cannulated drill was then used to create the narrow tunnel in an outside‐in fashion. Subsequently, a flip drill (Metal FlipDrill, Delta) was employed under arthroscopic guidance to create the socket from the inside out, with meticulous care taken to preserve a narrow tunnel segment of at least 7 mm. The graft was introduced into the bone tunnel, and the femoral side suspensory button was tightened when the pulling wire indicated a ‘flipping’ sensation. The tibial side suspensory button was tightened under direct visualization at the tibial incision. In our most recent protocol, a preloaded butterfly button (AC Bridge, SINOAR) was applied at the tibial tunnel aperture, beneath the adjustable‐loop button, for patients with osteoporosis or those deemed at high risk of cortical breach, to augment fixation strength and distribute the load. The knee joint was repetitively flexed while tightening both suspensory buttons until arthroscopic examination confirmed adequate graft tension, with no intercondylar notch impingement and a full range of motion in the knee joint.

Postoperative rehabilitation follows a standardized, phased protocol supervised by professional physical therapists. Within 24 h after surgery, training includes full knee extension exercises, isometric contraction of the quadriceps, and three‐dimensional ankle pump movements; after 48 h, closed‐chain eccentric training of the hamstrings and patellofemoral joint mobilization exercises are initiated. At 2 weeks postoperatively, the knee joint is flexed to 120°. Partial weight‐bearing is permitted at 1 month postoperatively, and full weight‐bearing is allowed at 2 months. Appropriate non‐contact sports can be conducted at 9 months, and exercise intensity is gradually increased after 1 year.

### Radiographic Assessment

Standardized anteroposterior (AP) radiographs of the knee were routinely obtained preoperatively, immediately postoperatively (typically within 48 h), and at 1, 3, 6, 12, and 24 months postoperatively. When clinically indicated by patient symptoms, additional advanced imaging—such as three‐dimensional computed tomography (3D‐CT) or magnetic resonance imaging (MRI)—was performed to complement the radiographic assessment. During the early postoperative period (typically within the first 6 months), standardized AP radiographs were acquired with the patient in a supine position to ensure stability, minimize joint loading, and reduce motion artifacts. The technical specifications were as follows: (1) Patient Positioning: Supine with the knee in full extension, slight internal rotation of the foot, and the popliteal fossa in contact with the detector. (2) Central Beam: Directed vertically to the inferior pole of the patella.

### Outcome measures

The following demographic data were collected: patient gender, age, height, weight, body mass index (BMI), injury side, operative time, status of meniscal injury, type of graft used, as well as the lengths and diameters of the femoral and tibial tunnels and the graft. Functional outcomes included: (1) Preoperative and postoperative (3, 6, 12, and 24 months) Lysholm Knee Score, International Knee Documentation Committee (IKDC) Subjective Score, and Tegner Activity Scale. (2) Knee stability assessment using the Ligs Digital Arthrometer (Innomotion Inc.) [17, 39]. The device simulated the Lachman test by measuring continuous displacement changes under incremental loads. Side‐to‐side differences (SSD) in anterior tibial translation were calculated at 80 N, 120 N, and 150 N of applied force (Figure 1b,c), quantifying anteroposterior laxity to evaluate stability preoperatively and at 12 and 24 months postoperatively. AIT‐related complications and their clinical manifestations were assessed and recorded based on surgical procedures, clinical follow‐up, and imaging data. All data were recorded and collected collaboratively by two experienced orthopaedic specialists.

### Complication evaluation method

**Figure 2:**
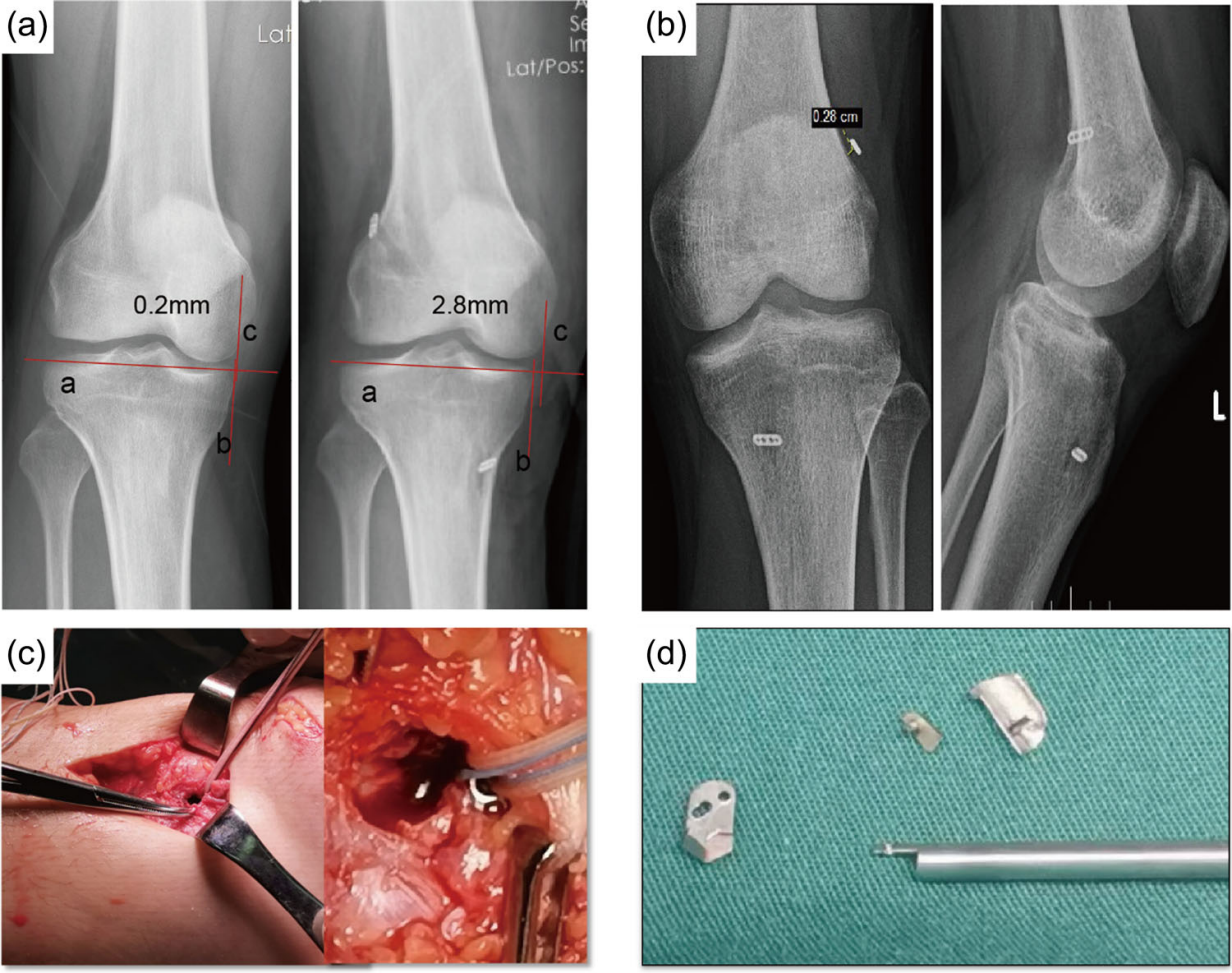
Radiographic and intraoperative views of AIT‐related complications. (a) Measurement of tibial lateral subluxation on AP radiographs. A line (Line a) was drawn tangent to the medial tibial plateau. A second line (Line b) was drawn tangent to the most medial edge of the tibial metaphysis, perpendicular to Line a. A third line (Line c) was drawn tangent to the most medial edge of the femoral metaphysis, perpendicular to Line a. An increase of (bc = 0.2 + 2.8 = 3.0) ≥2 mm in the distance between lines b and c postoperatively defines subluxation. (b) Postoperative AP and lateral radiographs showing malposition of the suspension button >2 mm from the lateral femoral cortex. (c) A cortical breach at the tibial tunnel exit. (d) A broken flip drill bit was retrieved during surgery. ACLR, anterior cruciate ligament reconstruction; AIT, all‐inside technique; AP, anteroposterior.

Based on the descriptive and measurement criteria established for tibial anterior subluxation, tibial lateral subluxation (Figure 2a) was radiographically defined as a postoperative lateral displacement of the tibia relative to the femur ≥ 2 mm compared to preoperative standardized AP radiographs [32, 40]. The measurement was performed on a standardized AP radiograph: A line (Line a) was drawn tangent to the medial tibial plateau. A second line (Line b) was drawn tangent to the most medial edge of the tibial metaphysis, perpendicular to Line a. A third line (Line c) was drawn tangent to the most medial edge of the femoral metaphysis, perpendicular to Line a. The accuracy of this radiographic measurement is highly dependent on the consistent identification of anatomical landmarks across all compared time points. For Line c, the medial edge of the femoral metaphysis is recommended as the primary landmark due to its relatively straight cortical contour. If the metaphyseal edge is ambiguous, the femoral intercondylar notch roof is a reliable alternative. The critical principle is to maintain consistency by using the same chosen landmark (either the metaphyseal edge or the intercondylar notch) for all pre‐ and post‐operative comparisons within a single patient. The horizontal distance between Line b and Line c was measured in millimetres (as illustrated in our Figure 2a). The difference in this distance between the postoperative and preoperative radiographs was calculated. An increase of ≥2 mm was defined as lateral subluxation. In this study, patients were assigned to the subluxation group through comparison of immediate postoperative (within 48 hours, non‐weight‐bearing) and preoperative AP radiographs, based on the identification of lateral tibial displacement relative to the femur. The subsequent resolution of the subluxation, together with knee function and stability, was systematically assessed during the follow‐up period.

Malposition of the suspensory button was classified according to the system described by Arthur et al. [2] as follows: Type I, where the button is fully contained within the bone tunnel; Type II, where it is partially within the tunnel; Type III, characterized by a gap of >2 mm between the button and the lateral femoral cortex; and Type IV, defined by a distance of ≥10 mm from the cortex, typically positioning the button over the iliotibial band.

### Statistical analysis

Statistical analysis was performed using SPSS 26.0 (IBM). Normality of quantitative variables was assessed with the Shapiro‐Wilk test. For normally distributed data, comparisons between groups were analyzed using Student's *t*‐test, and data were expressed as mean ± standard deviation (SD). For non‐normally distributed data, the Mann‐Whitney *U* test was used for group comparisons, with results expressed as the median and interquartile range (*M*, [Q1–Q3]). Categorical variables were presented as frequencies (%) and analyzed using chi‐square tests. To minimize confounding bias and address baseline imbalances between the complication and non‐complication groups, a propensity score matching (PSM) model was implemented to adjust for baseline differences. The following variables were included as covariates in the propensity score calculation: sex, age, height, weight, BMI, injury side, meniscal pathology, graft type, femoral/tibial tunnel lengths, and graft length/diameter (Table S1). Operative time was intentionally excluded from the propensity score model as it is directly influenced by the occurrence and management of complications. Nearest‐neighbor matching was performed at a 1:2 ratio (complication vs. non‐complication groups) with a caliper width of 0.2. Group comparisons were performed using independent *t*‐tests, with *p* values and 95% confidence intervals (CIs) reported. Statistical significance was set at *p* < 0.05.

## RESULTS

**Figure 3:**
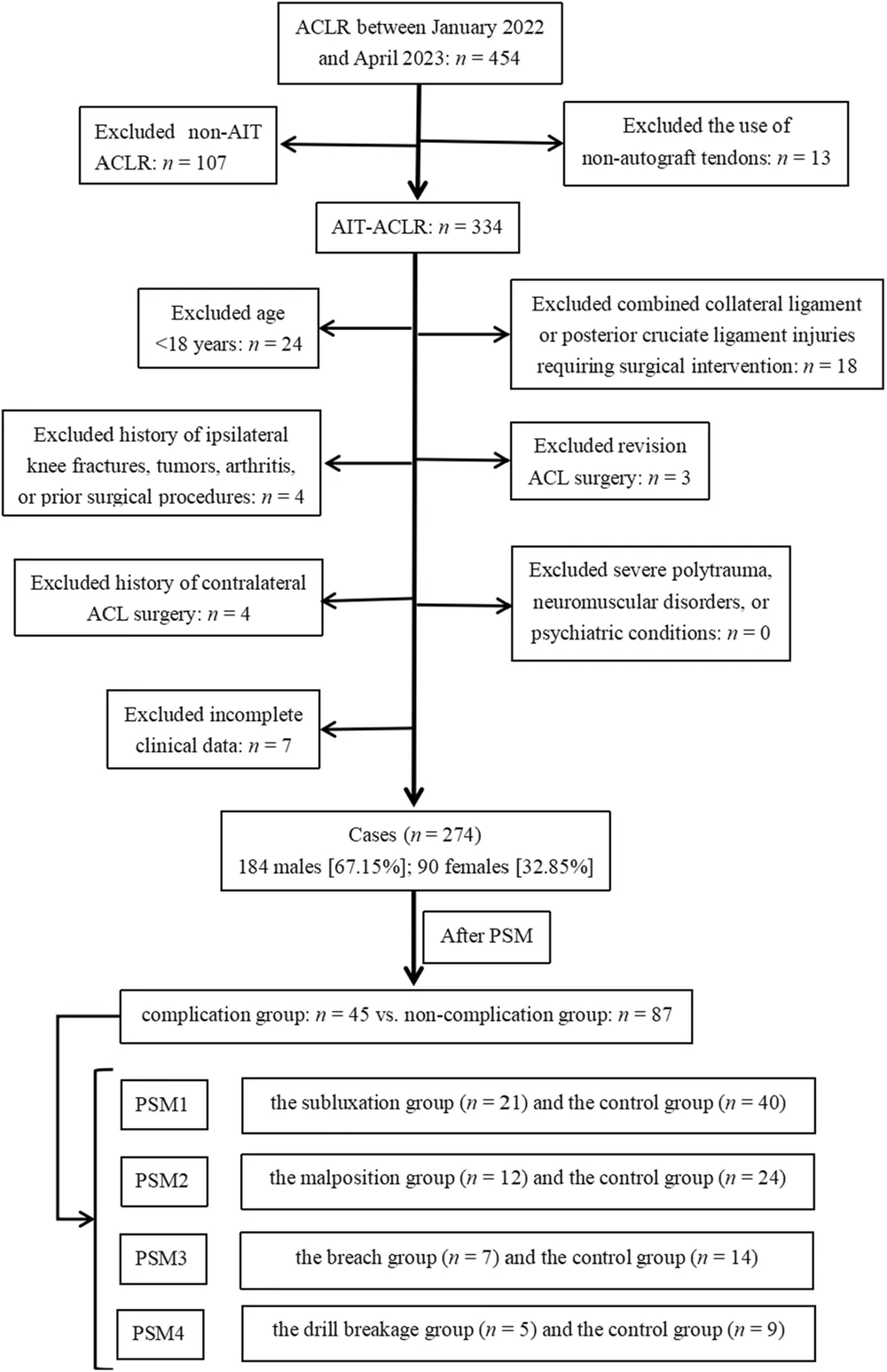
Patient selection flowchart for the PSM. The diagram illustrates the derivation of the final study cohort. From 274 initially enroled patients who underwent AIT‐ACLR, 45 with complications were exactly matched to 87 without complications from a pool of 229 eligible patients, after the exclusion of 3 unmatched controls. ACLR, anterior cruciate ligament reconstruction; AIT, all‐inside technique; PSM, propensity score matching.

A total of 274 patients (184 males [67.2%]; 90 females [32.8%]) were included in the analysis (Figure 3). The cohort had a mean age of 29.7 ± 8.2 years (range, 18–58 years) and a postoperative follow‐up duration of 29.6 ± 4.5 months (range, 24–39 months). The average length of the tendon graft was 64.8 ± 1.4 mm (range: 58.0‐67.0 mm), with a diameter of 8.7 ± 0.5 mm (range: 8.0–10.0 mm). Intraoperative findings revealed concomitant meniscal injuries in 227 patients and articular cartilage lesions in 16 patients. For the management of meniscal pathology, 170 patients underwent suture repair and 68 underwent meniscoplasty. All 16 cases of articular cartilage lesions, which presented with surface degeneration or a lesion depth of less than 50% (International Cartilage Repair Society [ICRS] grade ≤ 2), were addressed with chondroplasty.

To contextualize the AIT‐specific technical complications, the incidence of conventional postoperative complications was systematically recorded in the entire cohort. These included postoperative knee stiffness—defined as deficient knee flexion (active flexion < 90° or passive flexion < 120°) at the 1‐month postoperative follow‐up, mandating intervention—which occurred in 6 patients (2.2%); superficial surgical site infection (requiring antibiotic therapy) in 2 patients (0.7%); wound healing complications requiring prolonged wound care in 4 patients (1.5%); and no cases of graft failure (defined as symptomatic instability with confirmed rupture on MRI or arthroscopy). In contrast, 45 patients (16.4%) experienced complications classified as AIT‐related, including tibial lateral subluxation (*n* = 21, 7.7%), femoral suspensory button malposition (*n* = 12, 4.4%), cortical breach at the tibial tunnel exit (*n* = 7, 2.6%), and breakage of the flip drill bit (*n* = 5, 1.8%). Propensity score matching yielded a final cohort of 132 patients for comparative analysis, comprising 45 with complications and 87 without, following the exclusion of 3 unmatched individuals. In this matched cohort, baseline demographics were well‐balanced between groups, except operative time, which was significantly longer in the complication group (*p* = 0.035, Table 1).

**Table 1:** Demographic data after propensity score matching of both groups.

Variables	Non‐com group (*n* = 87)	Com group (*n* = 45)	*p* value	SMD
Sex, *n* (%)			0.60	
Male	54 (62.1)	30 (66.7)		0.09
Female	33 (37.9)	15 (33.3)		−0.09
Age, year^a^	29.2 ± 8.6	29.4 ± 7.4	0.87	0.03
Height, m^a^	1.7 ± 0.1	1.7 ± 0.1	0.68	0.08
Weight, kg^a^	72.6 ± 13.9	72.5 ± 13.2	0.97	−0.01
BMI, kg/m^2^ a	24.5 ± 3.5	24.3 ± 3.3	0.73	−0.07
Injury side, *n* (%)			0.87	
Left	40 (46.0)	20 (44.4)		−0.03
Right	47 (54.0)	25 (55.6)		0.03
Operative time, min^a^	94.4 ± 18.0	100.7 ± 17.9	0.03*	‐
Meniscal pathology, *n* (%)			0.90	
Normal	18 (20.7)	7 (15.6)		−0.14
Medial tear	14 (16.1)	7 (15.6)		−0.02
Lateral tear	33 (37.9)	19 (42.2)		0.09
Medial and lateral tear	22 (25.3)	12 (26.7)		0.03
Graft type, *n* (%)			0.79	
ST	54 (62.1)	29 (64.4)		0.05
ST + G	33 (37.9)	16 (35.6)		−0.05
Femoral tunnel, mm^b^	20.0 (20.0, 20.0)	20.0 (20.0, 20.0)	0.42	−0.09
Tibial tunnel, mm^b^	25.0 (25.0, 25.0)	25.0 (25.0, 25.0)	0.76	−0.02
Graft length, mm^b^	65.0 (65.0, 66.0)	65.0 (65.0, 65.0)	0.59	−0.08
Graft diameter, mm^b^	9.0 (8.0, 9.0)	9.0 (8.0, 9.0)	0.81	0.01

Abbreviations: BMI, body mass index; Com, complication; G, gracilis; non‐com, non‐complication; SMD, standardized mean difference; ST, semitendinosus.

^a^ Data expressed as mean ± standard deviation (SD).

^b^ Data expressed as median ((P_25_, P_75_).

* Statistically significant (*p* ˂ 0.05).

**Figure 4:**
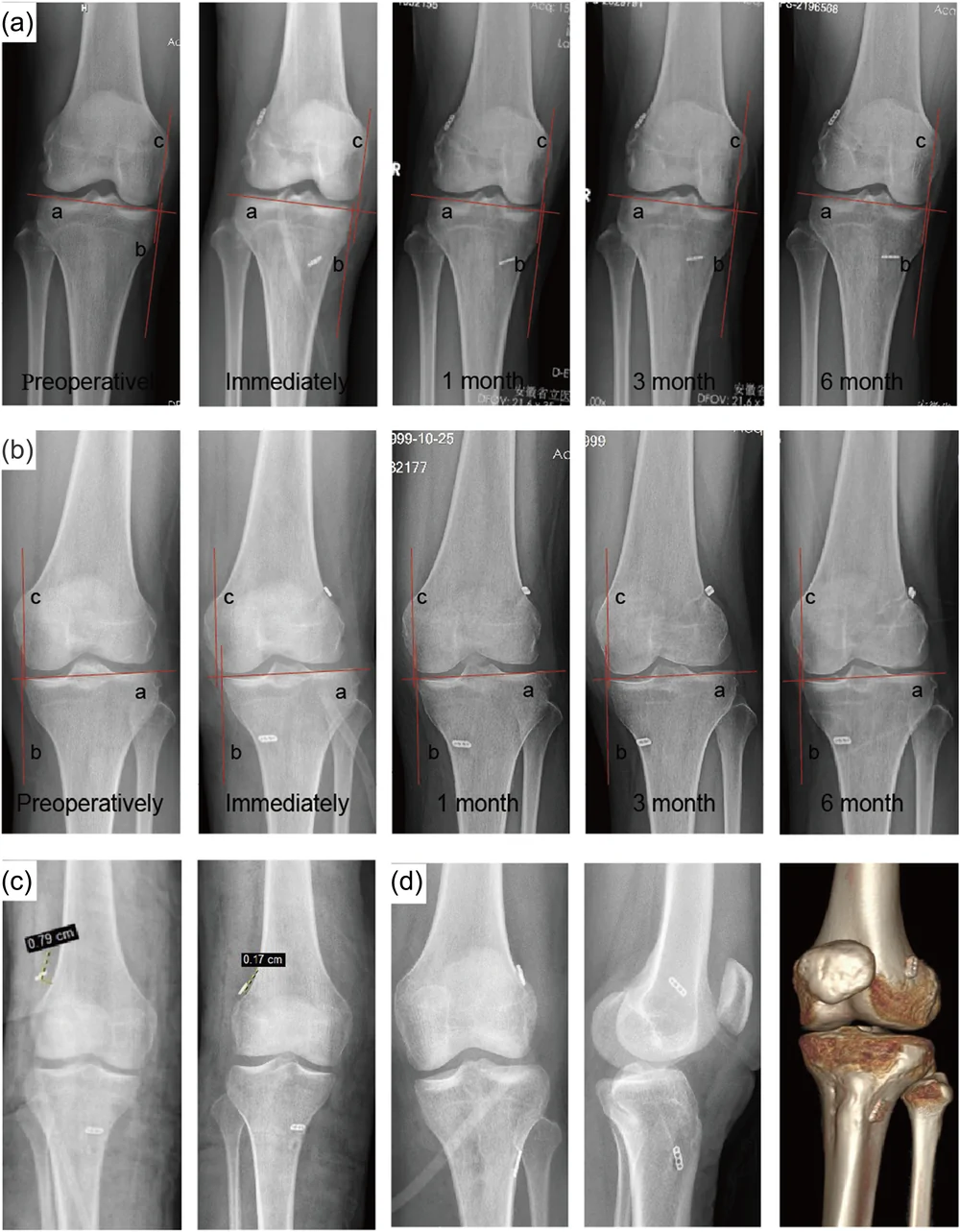
Representative radiographic courses following AIT‐ACLR. (a) Serial AP radiographs of a 25‐year‐old male showing transient tibial lateral subluxation on immediate postoperative view with spontaneous resolution by 1 month. (b) A 23‐year‐old female demonstrated a similar transient subluxation pattern that resolved completely within 3 months. (c) An 18‐year‐old female requiring immediate revision for severe femoral button malposition (left) with successful repositioning confirmed on post‐revision radiograph (right). (d) Postoperative AP and lateral radiographs and a 3D‐CT reconstruction of a 23‐year‐old female, demonstrating the salvage transverse tunnel technique for cortical breach management. ACLR, anterior cruciate ligament reconstruction; AIT, all‐inside technique; AP, anteroposterior; 3D‐CT, three‐dimensional computed tomography.

**Figure 5:**
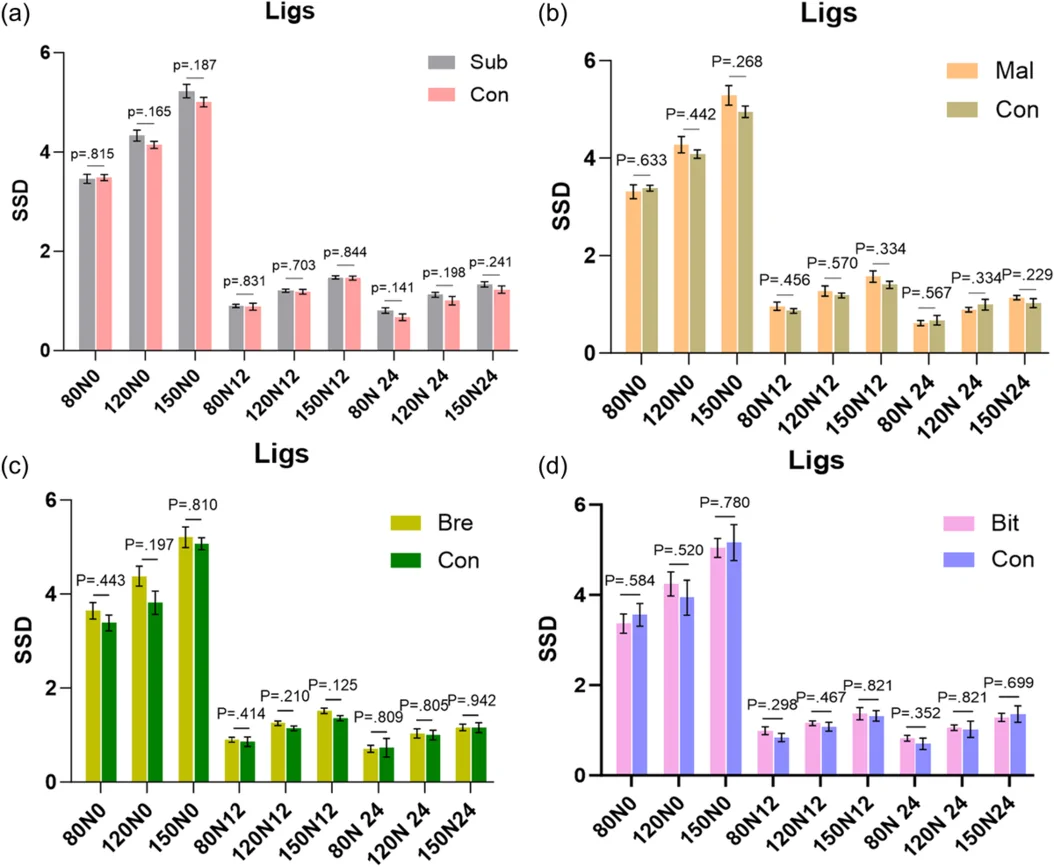
Knee stability outcomes are comparable between patients with and without complications. SSD in anterior tibial translation under 80, 120, and 150 N loads are shown for the complication subgroups and their PSM controls. (a) Tibial lateral subluxation group. (b) Femoral suspensory button malposition group. (c) Cortical breach at the tibial tunnel exit group. (d) Breakage of the flip drill bit group. The absence of significant differences at 12 and 24 months postoperatively demonstrates that properly managed complications do not compromise mid‐term stability. Bit, breakage of the flip drill bit; Bre, cortical breach at the tibial tunnel exit; Con, control; Mal, femoral suspensory button malposition; PSM, propensity score matching; SSD, side‐to‐side differences; Sub, tibial lateral subluxation; 80N0: 80 N before surgery; 80N12: 80 N at 12 months after surgery; 80N24: 80 N at 24 months after surgery.

Tibial lateral subluxation (Figure 2a) had an average displacement of 2.57 mm (range: 2.0–3.1 mm), and no specific intervention was performed for these patients. During the postoperative follow‐up, 4 cases (19.1%) demonstrated resolution of subluxation at 1 month postoperatively (Figure 4a), while 17 cases (80.9%) recovered by 3 months postoperatively (Figure 4b). Following PSM, there were no statistically significant differences in knee function and stability between the subluxation group (*n* = 21) and the control group (*n* = 40) at 3, 6, 12, and 24 months postoperatively (Tables 2, 3, 4 PSM1, Figure 5a). Only Type III suspensory button malposition (12 cases, 4.4%)—defined as a > 2 mm visible gap between the button and femoral cortex on anteroposterior radiographs (Figure 2b)—was observed in this study. Among these, one case (8.3%) had a displacement of 7.6 mm. It underwent immediate revision postoperatively (Figure 4c). After PSM, there were no statistically significant differences in knee function and stability between the malposition group (*n* = 12) and the control group (*n* = 24) at 3, 6, 12, and 24 months (Tables 2, 3, 4 PSM2, Figure 5b). The remaining 11 cases (91.7%) exhibited an average displacement of 2.5 mm (range: 2.1–3.0 mm) and received no specific treatment. Cortical breach at the tibial tunnel exit (Figure 2c) occurred in 7 cases (2.6%), all of which were managed by creating a 4.5 mm bone tunnel from medial to lateral, deep to the tibial tubercle. The tibial suspensory button was then secured from the medial tibial aspect to the thicker lateral cortical region under tension (Figure 4d). Post‐PSM, there were no statistically significant differences in knee function and stability between the breach group (*n* = 7) and the control group (*n* = 14) at 3, 6, 12, and 24 months postoperatively (Tables 2, 3, 4 PSM3, Figure 5c). Breakage of the flip drill bit (Figure 2d) occurred in 5 cases (1.8%) during the drilling of the tibial tunnel, without causing any damage to the bone tunnel. After PSM, no statistically significant differences in knee function and stability were found between the drill breakage group (*n* = 5) and the control group (*n* = 9) at 3, 6, 12, and 24 months postoperatively (Tables 2, 3, 4 PSM4, Figure 5d).

**Table 2:** Lysholm^a^ knee score in groups at 3, 6, 12, and 24 months postoperatively.

Variables	PSM1			PSM2			PSM3			PSM4		
	Sub Group	Con Group	*p* value	Mal Group	Con Group	*p* value	Bre Group	Con Group	*p* value	Bit Group	Con Group	*p* value
	(*n* = 21)	(*n* = 40)	(95%CI)	(*n* = 12)	(*n* = 24)	(95%CI)	(*n* = 7)	(*n* = 14)	(95%CI)	(*n* = 5)	(*n* = 9)	(95%CI)
Preoperative	52.1 ± 5.0	53.2 ± 9.3	0.57 (−4.8, 2.6)	52.8 ± 5.1	58.0 ± 9.0	0.07 (−10.9, 0.5)	53.3 ± 7.9	52.9 ± 11.6	0.94 (−7.9, 8.7)	53.2 ± 5.7	53.2 ± 9.5	0.99 (−8.3, 8.3)
3 months	73.7 ± 5.7	75.4 ± 5.4	0.24 (−4.4, 1.1)	75.4 ± 3.9	74.6 ± 3.4	0.51 (−1.7, 3.3)	68.1 ± 7.9	71.6 ± 7.3	0.33 (−10.7, 3.7)	76.0 ± 5.7	74.8 ± 2.9	0.60 (−3.6, 6.0)
6 months	84.3 ± 6.3	86.5 ± 6.2	0.20 (−5.6, 1.2)	85.4 ± 5.1	87.6 ± 7.7	0.38 (−7.1, 2.7)	84.0 ± 4.9	84.6 ± 7.6	0.86 (−6.5, 5.3)	85.2 ± 2.6	87.6 ± 2.6	0.13 (−5.6, 0.8)
12 months	89.9 ± 6.6	89.8 ± 7.2	0.95 (−3.7, 3.9)	90.7 ± 5.9	92.3 ± 5.7	0.43 (−5.7, 2.5)	89.0 ± 4.2	89.5 ± 9.2	0.89 (−7.6, 6.6)	92.0 ± 4.0	94.4 ± 2.0	0.15 (−5.7, 0.9)
24 months	91.7 ± 4.9	91.2 ± 5.0	0.73 (−2.3, 3.3)	92.2 ± 3.8	91.4 ± 4.1	0.59 (−2.1, 3.7)	91.3 ± 3.9	90.2 ± 6.9	0.71 (−4.4, 6.6)	92.2 ± 5.8	93.6 ± 3.8	0.61 (−5.9, 3.1)
* p* * Value	＜0.001	＜0.001		＜0.001	＜0.001		＜0.001	＜0.001		＜0.001	＜0.001	

Abbreviations: Bit, breakage of the flip drill bit; Bre, cortical breach at the tibial tunnel exit; CI, confidence interval; Con, control; Mal, femoral suspensory button malposition; PSM, propensity score matching; Sub, tibial lateral subluxation.

^a^ Data expressed as mean ± standard deviation (SD).

* Significant compared with preoperative values.

**Table 3:** IKDC^a^ subjective score in groups at 3, 6, 12, and 24 months postoperatively.

Variables	PSM1			PSM2				PSM3			PSM4		
	Sub Group	Con Group	*p* value	Mal Group		Con Group	*p* value	Bre Group	Con Group	*p* value	Bit Group	Con Group	*p* value
	(*n* = 21)	(*n* = 40)	(95%CI)	(*n* = 12)		(*n* = 24)	(95%CI)	(*n* = 7)	(*n* = 14)	(95%CI)	(*n* = 5)	(*n* = 9)	(95%CI)
Preoperative	41.6 ± 3.9	43.9 ± 8.9	0.18 (−5.6, 1.0)	42.3 ± 4.1		46.3 ± 9.4	0.08 (−8.5, 0.5)	42.4 ± 2.8	43.6 ± 12.3	0.74 (−9.8, 7.4)	41.6 ± 3.4	42.6 ± 8.7	0.82 (−8.5, 6.5)
3 months	61.6 ± 2.7	60.4 ± 8.4	0.40 (−1.7, 4.1)	61.0 ± 5.8		63.6 ± 11.2	0.45 (−9.4, 4.2)	56.4 ± 3.2	60.4 ± 11.4	0.25 (−10.9, 2.9)	60.8 ± 2.2	61.7 ± 8.3	0.77 (−6.8, 5.0)
6 months	72.3 ± 5.6	73.3 ± 4.9	0.47 (−3.7, 1.7)	74.8 ± 4.7		73.1 ± 3.5	0.57 (−2.3, 4.1)	72.4 ± 5.8	73.8 ± 4.8	0.58 (−6.2, 3.4)	73.0 ± 1.7	76.3 ± 4.2	0.12 (−7.5, 0.9)
12 months	81.3 ± 7.6	82.3 ± 6.2	0.59 (−4.7, 2.7)	83.7 ± 7.7		85.9 ± 5.5	0.32 (−6.7, 2.3)	85.4 ± 8.0	82.8 ± 4.3	0.33 (−2.8, 7.8)	88.6 ± 7.9	86.7 ± 3.6	0.54 (−4.3, 8.1)
24 months	90.5 ± 3.1	90.5 ± 3.6	0.94 (−1.8, 1.8)	89.5 ± 3.3		89.6 ± 3.4	0.95 (−2.7, 2.5)	89.4 ± 2.8	90.1 ± 3.4	0.63 (−3.9, 2.5)	90.4 ± 0.8	89.6 ± 3.2	0.63 (−2.6, 4.2)
*p* * Value	＜0.001	＜0.001		＜0.001		＜0.001		＜0.001	＜0.001		＜0.001	＜0.001	

Abbreviations: Bit, breakage of the flip drill bit; Bre, cortical breach at the tibial tunnel exit; CI, confidence interval; Con, control; Mal, femoral suspensory button malposition; PSM, propensity score matching; Sub, tibial lateral subluxation.

^a^ Data expressed as mean ± standard deviation (SD).

* Significant compared with preoperative values.

**Table 4:** Tegner^a^ activity scale in groups at 3, 6, 12, and 24 months postoperatively.

Variables	PSM1			PSM2				PSM3			PSM4		
	Sub Group	Con Group	*p* value	Mal Group		Con Group	*p* value	Bre Group	Con Group	*p* value	Bit Group	Con Group	*p* value
	(*n* = 21)	(*n* = 40)	(95%CI)	(*n* = 12)		(*n* = 24)	(95%CI)	(*n* = 7)	(*n* = 14)	(95%CI)	(*n* = 5)	(*n* = 9)	(95%CI)
Preoperative	1.7 ± 0.5	1.9 ± 0.5	0.33 (−0.5, 0.2)	1.9 ± 0.3		1.8 ± 0.6	0.27 (−0.1, 0.4)	1.7 ± 0.5	2.1 ± 0.6	0.20 (−1.0, 0.2)	1.6 ± 0.6	1.6 ± 0.7	0.91 (−0.7, 0.7)
3 months	3.1 ± 0.8	3.2 ± 0.7	0.38 (−0.5, 0.2)	3.3 ± 0.7		3.1 ± 0.8	0.35 (−0.2, 0.6)	3.1 ± 0.7	3.3 ± 0.8	0.70 (−0.8, 0.5)	3.0 ± 0.7	3.1 ± 0.9	0.82 (−0.9, 0.7)
6 months	4.4 ± 0.9	4.4 ± 0.8	0.89 (−0.4, 0.5)	4.8 ± 0.6		4.4 ± 0.7	0.15 (−0.1, 0.9)	4.3 ± 0.8	4.4 ± 0.7	0.66 (−0.7, 0.5)	4.2 ± 0.5	4.6 ± 0.5	0.23 (−1.0, 0.2)
12 months	5.7 ± 1.1	5.7 ± 0.9	0.89 (−0.5, 0.6)	5.9 ± 1.2		6.0 ± 0.6	0.75 (−0.8, 0.6)	5.6 ± 0.5	5.9 ± 0.7	0.27 (−0.8, 0.2)	5.8 ± 0.8	6.0 ± 0.7	0.64 (−0.9, 0.5)
24 months	6.0 ± 0.8	5.9 ± 0.9	0.91 (−0.4, 0.5)	5.8 ± 0.7		6.2 ± 0.7	0.15 (−1.0, 0.2)	5.9 ± 0.7	6.1 ± 0.9	0.59 (−0.9, 0.5)	6.2 ± 0.8	6.1 ± 0.8	0.85 (−0.7, 0.9)
*p* * value	＜0.001	＜0.001		＜0.001		＜0.001		＜0.001	＜0.001		＜0.001	＜0.001	

Abbreviations: Bit, breakage of the flip drill bit; Bre, cortical breach at the tibial tunnel exit; CI, confidence interval; Con, control; Mal, femoral suspensory button malposition; PSM, propensity score matching; Sub, tibial lateral subluxation.

^a^ Data expressed as mean ± standard deviation (SD).

* Significant compared with preoperative values.

## DISCUSSION

This study evaluated the spectrum of complications directly associated with the technical execution of AIT‐ACLR. In contrast to conventional ACLR complications (e.g., postoperative range of motion deficits, infection, or graft rupture), our analysis specifically focused on intraoperative technical complications inherent to the AIT procedure. Conventional complications occurred at a low frequency in our cohort and were managed promptly upon detection during early follow‐up. Furthermore, to isolate the impact of specific AIT‐related complications, potential confounding factors—including the presence of other concurrent complications—were controlled through rigorous group allocation strategies. Tibial lateral subluxation was the most frequent complication in our cohort, followed by malposition of the femoral suspensory button, cortical breach at the tibial tunnel exit, and breakage of the flip drill bit. To our knowledge, this is the first study to systematically describe the phenomenon of tibial lateral subluxation following AIT‐ACLR, noting its spontaneous resolution within 3 months postoperatively. Furthermore, to manage cortical breaches, we introduced and validated an innovative intraoperative transverse tunnel fixation technique as an effective salvage strategy.

To ensure a robust comparison, a PSM analysis was employed, which effectively balanced baseline covariates between groups. Although operative time—a variable influenced by complication management—was intentionally excluded from the matching model and was significantly longer in the complication group, no other demographic differences were observed after matching. Reassuringly, the PSM analysis confirmed that patients with appropriately managed complications showed no significant differences in mid‐term knee function or stability compared to matched controls. It is noteworthy that, although all confidence intervals for the comparisons included zero, some subgroup intervals were relatively wide. This indicates a degree of uncertainty in estimating the true effect size. These wide confidence intervals, coupled with the small sample sizes in certain subgroups, suggest that our study might have been underpowered. Consequently, there is a risk of Type II error (i.e., failing to detect a true difference if one exists). Therefore, while we can confidently state that no detrimental effects on mid‐term outcomes were observed, future studies with larger samples are warranted to definitively rule out more subtle differences.

The principal finding of this study is that when recognized and adequately addressed intraoperatively, these specific AIT‐related complications do not adversely affect mid‐term clinical outcomes. Nevertheless, their occurrence underscores the importance of technical vigilance, refined surgical execution, and the development of proactive prevention strategies to optimize the safety and efficacy of AIT‐ACLR further.

### Management of complications

#### Tibial lateral subluxation

Tibial lateral subluxation is a unique complication associated with AIT‐ACLR. AIT employs bilateral adjustable‐loop suspensory fixation devices, which allow for dynamic, sequential graft tensioning. This approach facilitates complete graft‐tunnel fill, thereby mitigating the adverse effects of synovial fluid infiltration on tendon‐to‐bone healing [6]. However, difficulties in graft insertion and a lack of effective monitoring methods for graft tension may lead to excessive tightening, resulting in lateral subluxation due to the application of excessive lateral forces to the tibia.

The tibial lateral subluxation identified in this study proved to be a self‐limiting condition that resolved spontaneously in the short term without the need for specific intervention and with no significant detrimental impact on postoperative knee function or stability. Its transient nature may be attributed to the dynamic process of graft adaptation and progressive tensioning that occurs during the early phases of rehabilitation and protected weight‐bearing. Although this phenomenon is relatively benign when weighed against the substantial risks associated with revision surgery, such as graft failure or infection, its recognition remains important to avoid unnecessary concern or intervention. Future studies are warranted to investigate whether such subluxation contributes to long‐term graft loosening or failure, as well as to elucidate the potential effects of high graft elongation rates on micromotion at the bone‐tendon interface [21]. To prevent intraoperative tibial lateral subluxation, a more standardized preventive approach involves drilling appropriately sized bone tunnels based on the dimensions of the woven graft and marking the tunnel length on the graft itself. During graft insertion, both sides of the button should be pre‐tightened to achieve uniform tension. The knee should be flexed and extended to facilitate graft creep, followed by alternating tightening of the loop while visually monitoring the graft under arthroscopy. Once the graft reaches the appropriate position and optimal tension, the pulling of the loop suture should cease, and the suture should be secured with a knot. Additionally, caution should be exercised to avoid over‐tensioning the graft in patients with suspected systemic ligamentous laxity—the correlation between graft tensioning and tibial lateral subluxation warrants further investigation.

#### Femoral suspensory button malposition

Malposition of the suspensory button typically occurs at the femoral fixation site, which may be attributed to the inability to achieve direct visualization during femoral tunnel fixation (unlike the tibial side, which allows visualization via the graft harvest incision). This necessitates a subcutaneous ‘loop‐flipping’ manoeuvre, during which soft tissue interposition or entrapment at the femoral tunnel aperture may occur [26]. Notably, soft tissue beneath the suspensory button cannot serve as a reliable fixation platform, leading to button subsidence and malposition, which may result in graft loosening and secondary structural laxity [2, 36]. Additionally, buttons entrapped at the femoral tunnel aperture risk dislodgement, compromising suspensory fixation and inducing graft laxity. Some patients may develop lateral femoral subcutaneous pain [14]. Furthermore, incomplete graft tensioning during loop fixation can contribute to femoral cortical button malposition, exacerbating graft laxity. Intriguingly, excessive loop tensioning may paradoxically predispose to subluxation, suggesting that the quantification of optimal tensioning forces represents a critical area for future research in AIT‐ACLR.

According to the classification by Arthur et al. [2], only Type III suspensory button malposition was observed following AIT‐ACLR. The absence of Types I/II malposition in our cohort likely reflects the efficacy of intraoperative ‘flipping’ feedback in achieving cortical fixation. Mild malposition ( ≤ 3 mm) did not require special intervention, while severe malposition ( > 3 mm) necessitated revision. The single case of severe malposition (7.6 mm displacement) requiring immediate revision was attributed to soft tissue interposition of the suspensory button superficial to the lateral femoral cortex. The revision was performed within the same operation. After fluoroscopic confirmation, the graft was removed, and a new suspensory button was deployed through the original femoral tunnel under continuous fluoroscopic guidance to ensure direct cortical contact before final graft tensioning and tibial. In both cases, there was no significant impact on knee function and stability postoperatively.

To prevent severe intraoperative malposition, the following strategies are recommended: intraoperative fluoroscopy, open visualization, arthroscopic assessment, and graft marking [2, 18]. O'Brien et al. [27] utilized intraoperative mini C‐arm fluoroscopy to confirm cortical suspensory button positioning, and all malpositioned buttons were corrected. However, the use of these techniques must be balanced against the potential for increased surgical time and unnecessary radiation exposure. Open visualization is the most rapid and effective method, but requires additional incisions, potentially increasing trauma to the patient. One approach to arthroscopic visualization involves extending the femoral traction line to visualize the button as it is pulled out of the bone tunnel, allowing for real‐time adjustment against the femoral cortex. Alternatively, the arthroscope can be inserted through the lateral incision to directly observe the emergence and positioning of the cortical suspensory button for secure fixation [26, 35]. Arthur et al. [2] suggested that using intraoperative fluoroscopy or open visualization can reduce the incidence of cortical suspensory button. Additionally, graft marking is another effective technique; by marking points on the graft corresponding to the appropriate tunnel length, surgeons can monitor the position of the button as it is pulled into the tunnel. It is recommended to include additional markers on the traction line at the length corresponding to the narrower tunnel, ensuring that the button is adequately secured against the bone cortex [10].

#### Cortical breach at the tibial tunnel exit

Cortical breach at the tunnel aperture is one of the most undesirable intraoperative complications for surgeons [23]. Potential risk factors may include insufficient tunnel length or low bone density [11, 18, 22]. In our study, all cortical breaches occurred on the tibial side, which may be attributed to the technical characteristics of the AIT. Specifically, after tightening the femoral side suspensory button, if excessive force is applied while re‐tightening the tibial side button, a breach of the cortical bone at the tunnel exit may occur. To address intraoperative cortical breaches at the tunnel aperture, Herbort et al. [11] proposed a fixation method combining a cortical button with embedded screws. Mitchell et al. [22] suggested a combination of embedded screws and washers for suspension fixation, with the graft traction line tied beneath the washer. Tsung‐Yu Lin et al. [18] utilized titanium hollow screws and washers, securing the button through the washer and using it as a substitute for the breached cortical bone, thereby providing support. A more traditional method involves further flexing the knee and re‐drilling the bone channel.

In addition to these methods, we prepared a 4.5 mm bone tunnel from the deep layer of the tibial tuberosity outward, securing the tibial medial loop button against the thicker lateral cortex. When a cortical breach occurs at the tibial exit and the button is sunken into the cortical bone, the femoral suspensory button has already been tightened. Intraoperatively, it is impossible to pull the tendon introduced into the joint cavity out of the cavity for reweaving without loosening the tibial suspensory button. Therefore, intraoperatively, the tibial‐side tendon, together with the button, must be pushed into the joint cavity. The button is then loosened under arthroscopic visualization. During the loosening process, suture‐grasping forceps can be used to fix the button in the joint cavity through an auxiliary incision, and a needle holder is employed to loosen the loop slowly. Care should be taken to protect the loop suture during loosening to avoid damage. Afterward, consideration is given to transferring and fixing the button through an auxiliary tunnel. Relevant studies have analyzed the gradation characteristics of the microhardness of the proximal tibial cancellous bone [37]. We drilled a transverse tunnel using a 4.5 mm drill bit at the distal end of the original cut tunnel exit and fixed the button on the lateral tibial cortex. The longer, narrower bone tunnel and the elimination of the vertical cutting tension exerted by the tibial plate on the tibial cortex enable this technique to effectively minimize the risk of recurrent cortical rupture, with subsequent evaluations revealing no significant clinical differences or outcome deficiencies. Although management strategies varied, the treatment of cortical breaches at the tunnel aperture can result in prolonged operative time and transient postoperative pain.

To ensure sufficient fixation strength of the adjustable‐loop suspensory button and prevent cortical breach at the tibial tunnel exit, it is essential to establish a narrow tunnel segment with a minimum length of greater than 7 mm [21]. Therefore, tunnel angulation and length must be carefully tailored to patient anatomy. Reduced strength of the tibial cortical bone may also cause the tibial cortex to be cut and breached by the suspensory button under tensile force; therefore, the patient's bone quality should be evaluated preoperatively. For elderly patients with severe osteoporosis, the size of the button can be changed to distribute the contact stress between the button and the tibial cortical bone. If no satisfactory button is available, a transverse tunnel can be preoperatively drilled from the inside out at the distal end of the original tunnel exit, and the button can be pulled to the lateral side of the tibia for fixation. In addition to osteoporosis caused by aging, surgeons should also pay attention to acute bone loss resulting from reduced weight‐bearing after trauma [7]. In recent cases involving osteoporotic bone or high‐risk patients, a preloaded butterfly button (AC Bridge, SINOAR, China) was applied at the tibial tunnel aperture beneath the adjustable‐loop button. Additionally, the tibial button was placed transversely to balance stresses at the proximal and distal ends. Short‐term follow‐up demonstrated satisfactory outcomes with reduced breach incidence, though long‐term efficacy requires further validation.

#### Breakage of the flip drill bit

Flip drill bit breakage may result from multiple factors, including steep entry angles between the cutting blade and cortical bone, incomplete blade rotation causing uneven stress distribution, or accumulated fatigue from repeated use. Immediate arthroscopic evaluation of tunnel integrity and retrieval of fractured fragments are critical upon breakage. After the timely intervention, the drill bit should be replaced to continue the preparation of the tunnel. In our cohort, flip drill bit breakage (*n* = 5, 1.8%) did not compromise osseous tunnels or clinical outcomes. However, the potential risks associated with the inability to retrieve broken fragments and the possibility of tunnel breach remain. Therefore, it is essential to actively prevent the breakage of the flip drill bit during the procedure. Preventive measures include monitoring drill bit quality in high bone density patients (e.g., young athletes) and initiating blade rotation at constant speeds before drilling.

#### Limitations

This study has several limitations that should be acknowledged. First, its single‐centre, retrospective design may limit generalizability due to potential selection bias. Second, despite employing PSM, residual confounding from unmeasured variables cannot be excluded. Third, the subjective definitions of complications and the non‐exhaustive classification system (e.g., excluding graft injuries or tunnel malposition) could affect reproducibility. Fourth, low complication rates resulted in relatively small sample sizes within each subgroup, which limited the statistical power for these specific comparisons and may affect the validity of the results. Critically, the non‐significant findings in this study may reflect this limited power and should not be interpreted as evidence of equivalence. Fifth, the measurement of subluxation may involve a novel/unvalidated metric. Finally, the mid‐term follow‐up (mean 29.6 months) precludes assessment of long‐term outcomes. Future multicenter studies with larger cohorts and longer follow‐up ( > 5 years) are needed to validate these findings and better address rare AIT‐related adverse events.

## CONCLUSION

This study confirms that AIT‐ACLR improves knee function and stability. While specific intraoperative complications—tibial lateral subluxation, femoral button malposition, tibial cortical breach, and flip drill bit breakage—may occur, they do not adversely affect mid‐term outcomes when properly managed. Tibial subluxation typically resolves spontaneously, whereas other complications underscore the importance of technical vigilance. Our findings highlight that surgical proficiency, encompassing both preventive strategies and adept complication management, is crucial for optimizing outcomes with the all‐inside technique. However, it should be noted that these comparative analyses were limited by statistical power and should be considered exploratory and descriptive in nature. Consequently, future studies with prospective designs and larger sample sizes are required to validate these observations.

## AUTHOR CONTRIBUTIONS

Yang Tang and Dongxu Yan contributed to study design, data interpretation, manuscript writing, and revision. Liang Xu and Di Wu provided study materials and contributed to data collection and assembly. Jingyu Gao and Yuan Wu provided relevant references. Gang Yu performed data analysis and interpretation. Qichun Zhao and Chao Fang conceived the study, provided financial and administrative support, and revised the manuscript. All authors read and approved the final manuscript.

## CONFLICT OF INTEREST STATEMENT

The authors declare no conflicts of interest.

## ETHICS STATEMENT

The Institutional Review Board of The First Affiliated Hospital of University of Science and Technology of China (Anhui Provincial Hospital) (Approval No. 2024‐RE‐276).

## Supporting information

Supporting information.

## Data Availability

The data are available from the corresponding author on reasonable request.
