# Supplementary material for: Management and analysis of complications associated with all‐inside technique anterior cruciate ligament reconstruction: A propensity score‐matched study
Source: J Exp Orthop. 2026 Jan 8;13(1):e70569. doi: 10.1002/jeo2.70569 (PMC12780871; doi:10.1002/jeo2.70569)
Supplement: Supplementary file 1 — Supporting information. [file JEO2-13-e70569-s001.docx]

**Supplemenary Table S1** Demographic data before Propensity Score Matching of both groups

| Variables | Non-com Group  (*n* = 229) | Com Group  (*n* = 45) | *p* Value | SMD |
| --- | --- | --- | --- | --- |
| Sex, *n* (%) |  |  | 0.65 |  |
| male | 155(67.6%) | 30(66.7%) |  | 0.07 |
| female | 74(32.3%) | 15(33.3%) |  | -0.07 |
| Age, year ^a^ | 29.8± 8.5 | 29.4 ± 7.4 | 0.80 | -0.05 |
| Height, m ^a^ | 1.7 ± 0.1 | 1.7 ± 0.1 | 0.50 | -0.12 |
| Weight, kg ^a^ | 75.4 ± 13.5 | 72.5 ± 13.2 | 0.19 | -0.22 |
| BMI, kg/m^2 a^ | 25.1 ± 3.6 | 24.3 ± 3.3 | 0.21 | -0.22 |
| Injury side, *n* (%) |  |  | 0.31 |  |
| left | 121(52.9%) | 20(44.4%) |  | -0.17 |
| right | 108(47.1%) | 25(55.6%) |  | 0.17 |
| Meniscal pathology, *n* (%) |  |  | 0.52 |  |
| normal | 45(19.6%) | 7(15.6%) |  | -0.15 |
| medial tear | 49(21.3%) | 7(15.6%) |  | -0.11 |
| lateral tear | 74(32.3%) | 19(42.2%) |  | 0.23 |
| medial and lateral tear | 61(26.6%) | 12(26.7%) |  | -0.05 |
| Graft type, *n* (%) |  |  | 0.16 |  |
| ST | 156(68.1%) | 29(64.4%) |  | -0.22 |
| ST+G | 73(31.9%) | 16(35.6%) |  | 0.22 |
| Femoral tunnel, mm ^b^ | 20.0 (20.0, 20.0) | 20.0 (20.0, 20.0) | 0.88 | -0.08 |
| Tibial tunnel, mm ^b^ | 25.0 (25.0, 25.0) | 25.0 (25.0, 25.0) | 0.97 | -0.11 |
| Graft length, mm ^b^ | 65.0 (65.0, 65.0) | 65.0 (65.0, 65.0) | 0.86 | 0.02 |
| Graft diameter, mm ^b^ | 9.0 (8.0, 9.0) | 9.0 (8.0, 9.0) | 0.67 | 0.04 |

Abbreviations: BMI, body mass index; Com, complication; G, gracilis; Non-com, non-complication; SMD, Standardized Mean Difference; ST, semitendinosus.

^a^ Data expressed as mean ± standard deviation (SD)

^b^ Data expressed as median ((P_25_, P_75_)

*Statistically significant (*P* ˂ 0.05).
